# Old marker, new face: anti-RNP as a sentinel of coronary and peripheral endothelial dysfunction in systemic lupus erythematosus

**DOI:** 10.1136/lupus-2026-002125

**Published:** 2026-06-29

**Authors:** Amany M. Ebaid, Mona Rabie, Wedad Mahmoud Ghazy, Shaimaa Wageeh, Mohammad Eltahlawi, Doaa Alhssein Abo-alella, Wafaa Metwally, Basma Magdy Elkholy, Lobna I Kotb

**Affiliations:** 1 Rheumatology and Rehabilitation Department, Faculty of Human Medicine, Zagazig University, Zagazig, Egypt; 2 Cardiovascular Department, Faculty of human medicine, Zagazig university, zagazig, Egypt; 3 Medical Microbiology and Immunology Department, Faculty of human Medicine, Zagazig University, Zagazig, Egypt; 4 Dermatology, Venereology and Andrology Department, Faculty of Human Medicine, Zagazig University, Zagazig, Egypt

**Keywords:** Lupus Erythematosus, Systemic, Autoantibodies, Cardiovascular Diseases, Microcirculation

## Abstract

**Objective:**

To investigate the association between anti-ribonucleoprotein (anti-RNP) antibodies and endothelial dysfunction (ED) in patients with SLE and their ability to predict early ED manifestations, both coronary and peripherally.

**Methods:**

A cross-sectional study was conducted at a tertiary hospital involving 70 patients with SLE meeting American College of Rheumatology (ACR)/Systemic Lupus International Collaborating Clinics (SLICC) criteria. All study participants underwent a full clinical and laboratory evaluation, including testing for Cardiovascular Diseases serum anti-RNP antibodies. Conventional transthoracic 2-dimensional echocardiography, tissue Doppler imaging and a transthoracic dobutamine stress echocardiography to assess coronary microvasculature by measuring coronary flow velocity reserve (CFVR). Peripheral microvascular morphology was evaluated via nailfold dermoscopy. The primary outcome measures were the mean CFVR values and the presence of dermoscopic abnormalities (capillary drop-out, tortuosity and density) in relation to anti-RNP titres.

**Results:**

In 70 patients with SLE, those with high disease activity (n=45) exhibited significantly impaired cardiac function compared with low-activity patients (n=25), showing lower ejection fraction (EF) and CFVR, alongside higher Wall Motion Score Index (p<0.05). Anti-RNP levels correlated positively with Systemic Lupus Erythematosus Disease Activity Index (SLEDAI, r=0.653, p<0.001), erythrocyte sedimentation rate, proteinuria and Raynaud’s phenomenon. Significant negative correlations existed between anti-RNP and stress EF (r=−0.33), CFVR (r=−0.30), C3 and C4 (p<0.05). Furthermore, elevated anti-RNP levels were significantly associated with clinical manifestations such as myositis and avascular necrosis, as well as dermoscopic microvascular changes, including microhaemorrhage and capillary dropouts (p<0.05).

**Conclusions:**

High anti-RNP titres are significantly associated with subclinical coronary microvascular impairment and peripheral capillary architectural changes in SLE. These results suggest that anti-RNP acts as a sentinel marker for early ED, providing potential for improved cardiovascular risk stratification independent of traditional risk factors.

**WHAT IS ALREADY KNOWN ON THIS TOPIC**

- SLE is associated with endothelial dysfunction, which was found to cause early, subclinical damage to small blood vessels and increase cardiovascular risk. Yet, the specific role of anti-ribonucleoprotein (anti-RNP) antibodies in microvascular endothelial dysfunction was not well defined.

**WHAT THIS STUDY ADDS**

- High anti-RNP titres are directly associated with reduced coronary blood flow and structural damage to skin capillaries, even in patients without traditional risk factors. This confirms that these antibodies serve as a specific indicator of microvascular impairment in both the heart and peripheral vessels.

**HOW THIS STUDY MIGHT AFFECT RESEARCH, PRACTICE OR POLICY**

- The findings suggest that anti-RNP can be used as an early marker of endothelial dysfunction, enabling earlier identification of at-risk patients and more personalised cardiovascular screening and risk management. This could lead to changes in clinical practice in which antibody positivity guides the preventive heart care for patients with SLE.

## Introduction

 SLE is a multisystemic autoimmune disease characterised by a profound loss of self-tolerance to nuclear antigens and the subsequent production of pathogenic autoantibodies.^1^ While anti-double-stranded DNA (anti-dsDNA) and anti-Smith (anti-Sm) are established hallmarks of the disease, the clinical significance of anti-ribonucleoprotein (anti-RNP) (also commonly referred to as anti-U1-RNP) antibodies targeting the small 70 kDa U1 nuclear ribonucleoprotein complex has regained attention. Although anti-RNP autoantibodies remain a cardinal serologic marker for mixed connective tissue disease (MCTD), they are also frequently detected in patients with SLE.^2^

Endothelial cells are now recognised as key components of the immune system, subject to both involvement and modulation by innate and adaptive immune responses.^3^ Endothelial dysfunction (ED), which occurs due to the chronic and systemic inflammatory processes presented in SLE, is considered the crucial trigger for atherosclerosis and cardiovascular events, which are the most common causes of premature mortality in patients with SLE.^4 5^ Because ED is theoretically reversible, it is a potentially attractive target for preventive interventions against the development of microvascular-related events.^6^

The exact role of anti-RNP antibodies in the pathogenesis of autoimmune diseases remains unclear. The literature indicates that anti-RNP antibodies are associated with manifestations of vascular disorders, suggesting that these antibodies increase cytokine levels, including interleukin (IL)-1α, IL-1β, IL-6 and TNF-α.^7^ They also upregulate the expression of adhesion molecules, including intercellular adhesion molecule-1 (ICAM-1), E-selectin and Class II molecules on human pulmonary artery endothelial cells. These findings suggest that these autoantibodies may cause endothelial cell dysfunction and lead to proliferative vasculopathy.^8 9^

Taken together, these findings have led us to hypothesise that there may be a deeper connection between anti-RNP antibodies and ED in SLE and its microvascular-related manifestations, such as cardiovascular disease (CVD), Raynaud’s disease (RD), osteonecrosis (ON) and glomerulonephritis.

## Materials and methods

### Patient selection

A cross-sectional study was conducted on patients with SLE, selected by a simple random method from the Rheumatology and Rehabilitation Department, and in collaboration with the Medical Microbiology, Immunology Department, Cardiology Department and Dermatology Department, Zagazig University Hospitals. Assuming the frequency of anti-Smith antibody (anti-Sm) was 31.4% versus 1% in anti-RNP antibody positive versus negative. At 80% power and 95% CI, the estimated sample was 56 cases, Open Epi. The work has been conducted in accordance with the Code of Ethics of the World Medical Association (Declaration of Helsinki) for studies involving humans.

Patients were recruited at the Rheumatology outpatient clinics. They were considered eligible if they matched the following inclusion criteria: had SLE disease diagnosed according to Systemic Lupus International Collaborating Clinics (ACR/SLICC) classification criteria for SLE.^10^ Patients matching the following criteria were excluded: those who refused consent, had severe comorbidities (cancer, known cardiovascular illness or major risk factors for CVD), including high blood pressure (by history and clinical assessment making sure that blood pressure is normal), high cholesterol (by measurement of blood lipid profile), smoking (by history), diabetes (by history and measurement of fasting and postprandial blood glucose levels) or obesity (by assessment of anthropometric measure including weight and height to evaluate body mass index considering obesity more than 30), we also excluded patients who were diagnosed with any other autoimmune disease, like MCTD, systemic sclerosis or vasculitis and those who receive B blockers and lipid-lowering drugs. Finally, we were able to recruit 70 patients with SLE who matched the selection criteria.

### Assessment of disease activity

All patients were subjected to full history taking, general, musculoskeletal and systemic examinations. Disease activity was assessed using the Systemic Lupus Erythematosus Disease Activity Index (SLEDAI-2K).^11^ According to established disease activity categories^12^ scores were classified as no activity (0), low/mild activity (1-5) moderate activity (6–10), high activity (11–19) or very high activity (≥20). For comparative analysis, patients with no, low/mild or moderate disease activity were combined into the no/low/moderate disease activity group, whereas patients with high or very high disease activity were combined into the high/very high disease activity group.

A 5 mL of peripheral blood was obtained on plain blood collection vacutainer tubes from all participants. About 2 mL of the blood sample was used to perform haematological examination (complete blood picture, erythrocyte sedimentation rate (ESR)), and the remaining of the sample was allowed to clot for 10–20 min at room temperature and centrifuged at 2000 Revolution per minute (RPM) for 20 min to separate serum, serum was harvested and stored at −20°C until use for detection of C reactive protein (CRP), C3, C4 and autoantibodies: ANA, anti-dsDNA, anti-RNP.

Serum CRP, C3 and C4 levels were measured by nephelometry. CRP level is considered elevated if more than 6 mg/L,^13^ normal C3 ranges between 90 mg/dL and 180 mg/dL and normal C4 ranges between 10 mg/dL and 40 mg/dL.^14^

ANA and anti-dsDNA were investigated using indirect immunofluorescence assays. ANA Immunofluorescence assay (IFA) with Human Epithelial type 2 (HEp-2) cells was performed using kits (Bio-Rad Laboratories, Benicia, California, USA). The cut-off for ANA positivity was 1/40.^15^ Anti-dsDNA was detected by using Crithidia luciliae indirect immunofluorescence assay (CLIFT) commercial kit (Alphadia SA/NV, Belgium). A positive test was considered at a titre of 1:10 or above.^16^ Anti-RNP antibodies were detected using a quantitative ELISA kit (Alpha Diagnostic International, USA), serum levels of 25 U/mL or more are considered positive.^17^

### Conventional echocardiography

All study participants underwent conventional transthoracic 2-dimensional echocardiography, using a commercially available echocardiography system (GE VIVID E9 Ultrasound Machine equipped with a 1.5–3.6 MHz phased array probe). Left ventricular end-diastolic volume and end-systolic volume were used to calculate ejection fraction (EF), measured by modified biplane Simpson’s method according to the American Society of Echocardiography and European Association of Cardiovascular Imaging.^18^

Tissue Doppler imaging is used to measure the septal and lateral mitral annular velocity, early diastolic (e) septal mitral annular velocity and the ratio of early trans mitral flow velocity to early diastolic mitral annular velocity (E/e ratio).

### Dobutamine stress echocardiography (DSE)

All patients underwent a standard graded dobutamine infusion protocol. Dobutamine was administered intravenously starting at a dose of 5 mcg/kg/min, increasing at 3 min intervals to 10, 20, 30 and a maximum of 40 mcg/kg/min. Atropine (up to 1 mg) was administered if the target heart rate (85% of the age-predicted maximum) was not achieved. The rest, low-dose, peak and recovery images were compared in a four-division screen; standard 12-lead ECG and blood pressure monitoring were performed throughout the infusion and recovery periods (onlinesupplemental videos 1^3^). Wall Motion Score Index (WMSI) was calculated in each patient at baseline and peak stress, in a 4-point score as (normal=1, hypokinesia=2, akinesia=3, dyskinetic=4) in a 17-segment model of the left ventricle.^19^

### Coronary flow velocity assessment

Transthoracic Doppler echocardiography was used to visualise the left anterior descending coronary artery in the interventricular groove using a high-frequency transducer. Coronary flow velocity was recorded at baseline (rest) and during peak stress (DSE) using pulsed-wave Doppler.

### Hemodynamic calculations

The diastolic peak velocity (DPV) was measured from the Doppler signal at two points of time:

1. DPV-Rest: the peak diastolic velocity at baseline.
2. DPV-DSE: the peak diastolic velocity during maximum dobutamine stress.

The coronary flow velocity reserve (CFVR) was defined as the ratio between the hyperaemic peak and the basal peak diastolic coronary flow velocities.^20^ CFVR was calculated using the following formula: CFVR=DPV (DSE)/DPV (Rest).

A CFVR value was considered abnormal if it was below the prespecified clinical threshold (typically <2.0), indicating impaired coronary flow augmentation.^21^

### Nailfold capillaroscopy (NFC)

All participants underwent standardised NFC to evaluate peripheral microvascular abnormalities. To ensure haemodynamic stability, examinations were conducted in a temperature-controlled environment (20°C–25°C) following a 20 min acclimatisation period. Participants adhered to preprocedural constraints, including a 2-week avoidance of nail trauma (manicures/procedures) and a 6-hour abstinence from smoking and caffeine.

#### Procedure and instrumentation

Visual enhancement was achieved by applying an immersion medium (mineral oil) to the nailfold interface. Examinations were performed using a DermLite DL4 handheld dermoscope (polarised mode, 10× magnification). The nailfolds of the second through fifth digits on both hands were systematically evaluated, with representative digital images captured for each participant.^22^

#### Morphological parameters

Assessments focused on three primary categories of microvascular and periungual change:

1. Periungual features: cuticular fragmentation (ragged cuticles), periungual erythema and telangiectasia.
2. Microvascular architecture: capillary dilatation (ectasia) and microhaemorrhages (punctate or bushy).
3. Structural integrity: capillary dropout and the presence of avascular zones.

Each parameter was recorded as ‘present’ if observed in at least two fingers.

Standardisation and blinding to eliminate observer bias, all evaluations were performed by a single experienced dermatologist blinded to the participants’ clinical profiles, laboratory results and anti-RNP antibody status. All digital images were archived and underwent a secondary review to ensure interassessment consistency.

### Statistical analysis

Data analysis was performed using IBM SPSS Statistics, V.26 (IBM Corp, Armonk, New York, USA). Descriptive statistics for categorical variables are presented as frequencies and percentages. The normality of quantitative variables was assessed using the Shapiro-Wilk test. The Mann-Whitney U test was employed to compare independent groups for variables exhibiting non-normal distributions. The association between continuous variables was evaluated using Spearman’s rank-order correlation coefficient (ρ). A significance level of p≤0.05 was adopted for all statistical tests.

## Results

A total of 70 patients with SLE were included in this study: 45 had high disease activity and 25 had low disease activity. Baseline characteristics of the study population are shown in table 1.

**Table 1:** Baseline characteristics of the studied patients with SLE

	All patients (n=70)	High activity (n=45)	Low activity (n=25)	P value
Age (years)	30.93±7.73	31.5±7.5	30.0±8.0	0.44
Sex				
Male	8 (11.4%)	5 (11.1%)	3 (12%)	
Female	62 (88.6%)	40 (88.9%)	22 (88%)	0.91
Disease duration (years)	6.23±2.01	5.2±1.8	4.9±1.4	0.47
Clinical data				
Seizures	1 (1.4%)	1 (2.2%)	0 (0%)	0.45
Psychosis	1 (1.4%)	1 (2.2%)	0 (0%)	0.45
Vasculitis	5 (7.1%)	5 (11.1%)	0 (0%)	0.08
Arthritis	29 (41.4%)	21 (46.7%)	8 (32%)	0.23
Arthralgia	60 (85.7%)	43 (95.6%)	17 (68%)	**0.002**
Myositis	5 (7.1%)	5 (11.1%)	0 (0%)	0.45
Serositis	18 (25.7%)	16 (35.6%)	2 (8%)	**0.01**
Skin rash	34 (48.6%)	24 (53.3%)	10 (40%)	0.28
Alopecia	58 (82.9%)	42 (93.3%)	16 (64%)	**0.002**
Oral ulcer	46 (65.7%)	40 (88.9%)	6 (24%)	**<0.001**
Fever	13 (18.6%)	12 (26.7%)	1 (4%)	**0.02**
Discoid rash	4 (5.7%)	4 (8.9%)	0 (0%)	0.29
Raynaud’s disease	47 (67.1%)	39 (86.7%)	8 (32%)	**<0.001**
Nephritis	50 (71.4%)	43 (95.6%)	7 (28%)	**<0.001**
AVN	9 (12.9%)	9 (20%)	0 (0%)	**0.02**
Laboratory data				
WBCs	6.06±2.93	4.3±2.5	5.5±2.0	**0.04**
HB	10.58±1.57	9.8±1.2	11.5±1.0	**<0.001**
PLT	264.91±97.3	220±80	300±60	**<0.001**
24 hours protein	675 (100–4500)	900 (400–4500)	300 (100–800)	**<0.001**
CRP	5.5 (3.5–14.1)	6 (4–14.1)	4 (3.5–12)	0.231
ESR	41 (30.8–60.8)	55 (40–60.8)	30 (30.8–45)	**<0.001**
Immunological data				
Positive ANA	70 (100.0%)	45 (100%)	25 (100%)	1
Positive anti-dsDNA	48 (68.6%)	35 (77.8%)	13 (52%)	**0.03**
C3	88 (71–119)	75 (71–90)	100 (85–119)	**<0.001**
C4	13 (9–25)	10 (9–15)	15 (12–25)	**<0.001**
Anti-RNP	23 (6.7–29.8)	25 (10–29.8)	20 (6.7–28)	**0.04**
Disease activity				
SLEDAI score	10 (4–16.3)	14 (10–16.3)	6 (4–9)	**<0.001**
Medications				
Azathioprine+CCs	33 (47.1%)	21 (46.7%)	12 (48.0%)	0.9
MMF+CCs	25 (35.7%)	16 (35.6%)	9 (36.0%)	0.97
MTX+CCs	1 (1.4%)	1 (2.2%)	0 (0.0%)	0.45
Cyclophosphamide	6 (8.6%)	4 (8.9%)	2 (8.0%)	0.89
MMF+ciclosporin	5 (7.1%)	3 (6.7%)	2 (8.0%)	0.36
Dermoscopic findings*				
Ragged cuticle	64 (91.4%)	41 (91.1%)	23 (92.0%)	0.89
Periungual erythema	23 (32.9%)	14 (31.1%)	8 (32.0%)	0.94
Microhaemorrhage	8 (11.4%)	6 (13.3%)	2 (8%)	0.5
Capillary dropouts	15 (21.4%)	13 (28.9%)	2 (8%)	**0.04**
Periungual pigmentation	23 (32.9%)	15 (33.3%)	9 (36%)	0.82
Mild capillary dilatation	15 (21.4%)	11 (24.4%)	4 (16%)	0.41

Data were expressed as number %, mean±SD or median (range), p value is significance if ≤0.05.

* Same patient may have >1 finding.

ANA, anti-nuclear antibody; anti-dsDNA, anti-double-stranded DNA; Anti-RNP, anti-ribonucleic protein antibody; AVN, avascular necrosis; C, complement; CCs, steroid; CRP, C reactive protein; ESR, erythrocyte sedimentation rate; MMF, mycophenolate mofetil; MTX, methotrexate; SLEDAI, SLE Disease Activity Index.

**Figure 1:**
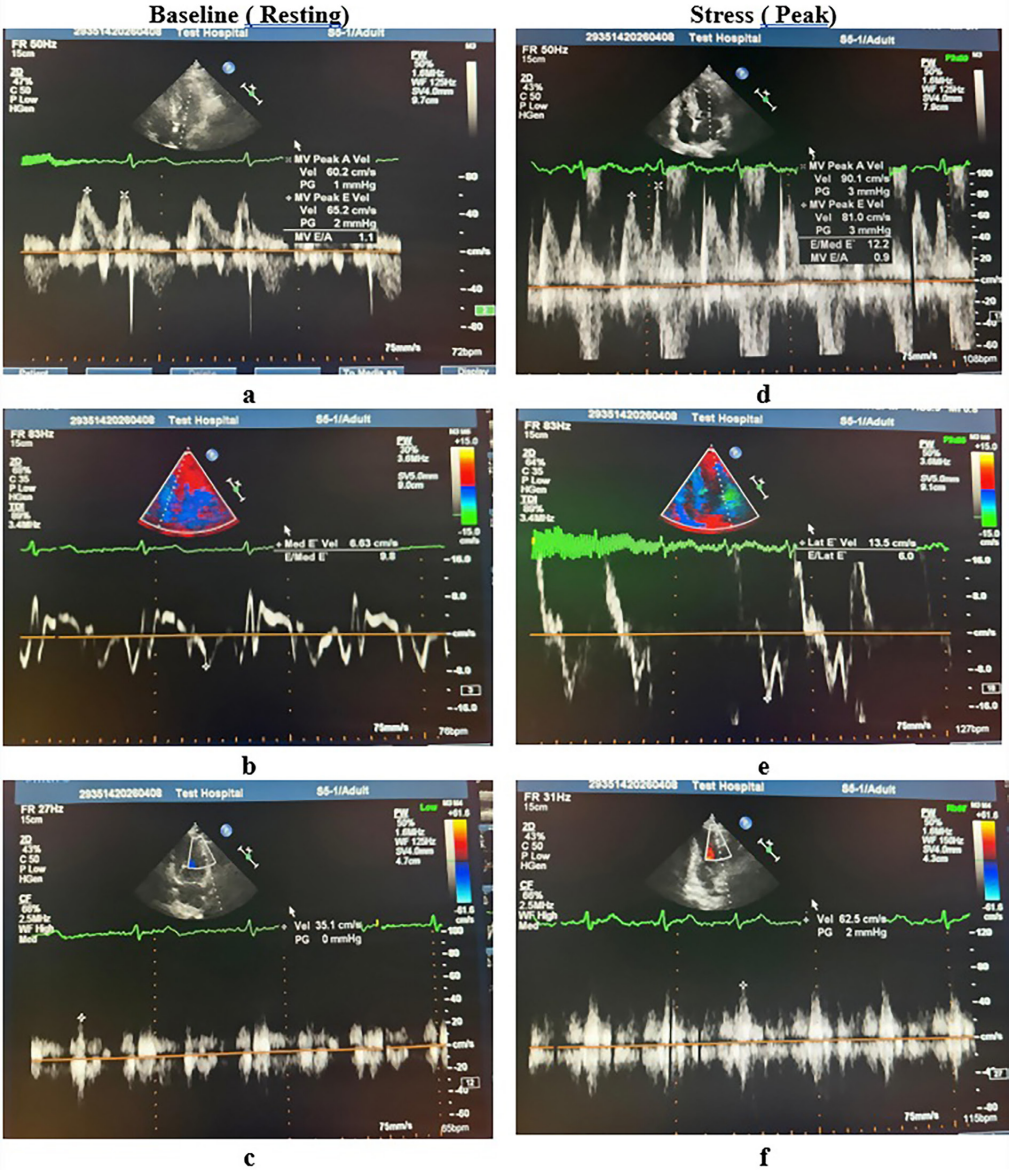
Dobutamine stress echocardiography during rest and stress. (**a**) Pulsed-wave Doppler of mitral inflow. At baseline, the E/A ratio is normal (>1.0). (**b**) TDI of the mitral annulus. Measurement of early diastolic myocardial velocity (**e’**) (baseline). (**c**) Pulsed-wave Doppler recording of the LAD coronary artery at the baseline stage. The distal LAD flow was identified in the anterior interventricular groove using colour Doppler guidance. The baseline peak diastolic velocity (bPDV) was measured at 35.1 cm/s. (**d**) Pulsed-wave Doppler recording of the mitral inflow at peak stress. (**e**) TDI of the mitral annulus during the peak stress stage. (**f**) Pulsed-wave Doppler recording of the distal LAD coronary artery during the hyperemic/peak stress stage. The Peak Diastolic Velocity (pPDV) was measured at 62.5 cm/s. LAD, left anterior descending; TDI, tissue Doppler imaging.

DES findings are illustrated in table 2. At rest, patients with high disease activity demonstrated significantly impaired cardiac functional parameters compared with those with low disease activity. Specifically, the high activity group showed lower EF (p=0.004), higher ratio of early transmitral flow velocity to early diastolic mitral annular velocity (E/e ratio) (p=0.004), higher WMSI (p<0.001) and higher DPV (p<0.001). No significant difference in resting heart rate was observed between the two groups (p=0.15). During stress DSE, patients with high SLE activity continued to exhibit significantly worse cardiac function. The high-activity group had lower EF (p<0.001), higher E/e ratio (p=0.003), higher WMSI (p<0.001) and higher DPV (p=0.008) than the low-activity group. In addition, CFVR was significantly reduced in the high-activity group (1.8±0.6) when compared with the low-activity group (2.3±0.5) (p<0.001) (figure 1).

**Table 2:** DSE findings in patients with SLE

	All patients (n=70)	High activity (n=45)	Low activity (n=25)	P value
Resting DSE				
Heart rate	78.38±9.05	78.5±10.2	75±8.5	0.15
EF	62.63±3.34	60±4	63±2.5	**0.004**
E/e	6.59±1.81	7.5±2	5.8±1.5	**0.004**
WMSI	1.21±0.18	1.32±0.16	1.08±0.11	**<0.001**
DPV	0.43±0.1	0.55±0.15	0.35±0.08	**<0.001**
Stress DSE				
Heart rate	142.9±5.9	138±6.5	147±5	**<0.001**
EF	76.4±3.1	72±4	79±2.5	**<0.001**
E/e	6.2±1.9	7±2.2	5.5±1.6	**0.003**
WMSI	1.36±0.24	1.50±0.22	1.12±0.14	**<0.001**
DPV	2.06±0.7	2.3±0.8	1.8±0.6	**0.008**
CFVR	2.06±0.7	1.8±0.6	2.3±0.5	**<0.001**

p value is significance if ≤0.05

CFVR, Coronary Flow Velocity Reserve; DPV, Diastolic Peak Velocity; DSE, Dobutamine Stress Echocardiography; E/e, ratio of early transmitral flow velocity to early diastolic mitral annular velocity; EF, Ejection Fraction; WMSI, Wall Motion Score Index.

**Figure 2:**
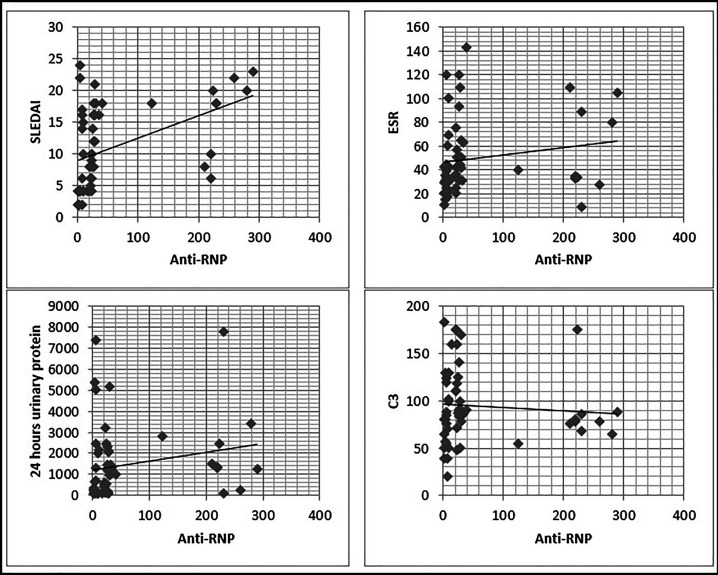
Correlation between anti-RNP and activity parameters among studied patients with SLE. anti-RNP, anti-ribonucleoprotein; SLEDAI, Systemic Lupus Erythematosus Disease Activity Index.

Table 3 shows the correlations between anti-RNP levels and various clinical, laboratory and DES parameters in the studied patients with SLE. Anti-RNP levels showed a strong positive correlation with SLEDAI. Significant positive correlations were also observed with ESR and 24-hour proteinuria. Conversely, anti-RNP levels exhibited significant negative correlations with haemoglobin, C3 and C4. Regarding cardiac functional parameters assessed by DSE, anti-RNP levels showed a significant negative correlation with stress EF and CFVR. However, no statistically significant correlations were observed between anti-RNP levels and other resting or stress DSE parameters, including heart rate, E/e ratio or DPV. (Some correlations are illustrated in figure 2).

**Table 3:** Correlation between anti-RNP and different parameters in patients with SLE

	Anti-RNP	
	r	P value
SLEDAI	0.653	**<0.001**
CRP	0.148	0.277
ESR	0.395	**0.003**
WBCs	−0.220	0.098
HB	−0.275	**0.042**
PLT	−0.192	0.135
24 hours protein	0.468	**0.001**
C3	−0.520	**<0.001**
C4	−0.311	**0.04**
Resting DES		
Heart rate	0.18	0.19
EF	−0.25	0.09
E/e	0.28	0.06
DPV	−0.19	0.16
Stress DES		
Heart rate	0.14	0.28
EF	−0.33	**0.03**
E/e	0.25	0.08
DPV	−0.22	0.11
CFVR	−0.30	**0.047**

p value is significance if ≤0.05

Anti-RNP, anti-ribonucleic protein; C, complement; CFVR, coronary flow velocity reserve; CRP, C reactive protein; DES, dobutamine stress echocardiography; DPV, diastolic peak velocity; E/e, ratio of early transmitral flow velocity to early diastolic mitral annular velocity; EF, ejection fraction; ESR, erythrocyte sedimentation rate; HB, haemoglobin; PLT, platelet; SLEDAI, SLE Disease Activity Index; WBCs, white blood cells.

Additional analyses were conducted (shown in online supplemental table 1) to explore the association between anti-RNP levels and various clinical manifestations of SLE. Anti-RNP levels were significantly higher in patients with avascular necrosis (AVN; median 29.8 vs 21.0, p=0.011), skin rash (median 24.6 vs 20.0, p=0.037) and oral ulcers (median 26.8 vs 9.0, p=0.029). Notably, the most substantial elevation was observed in patients with myositis, who exhibited median levels of 154.9 compared with 21.0 in those without the condition (p=0.010). Furthermore, a significant positive correlation was identified between anti-RNP levels and Raynaud’s phenomenon (RP, p=0.018). No statistically significant associations were found for the remaining clinical manifestations (all p>0.05).

**Figure 3:**
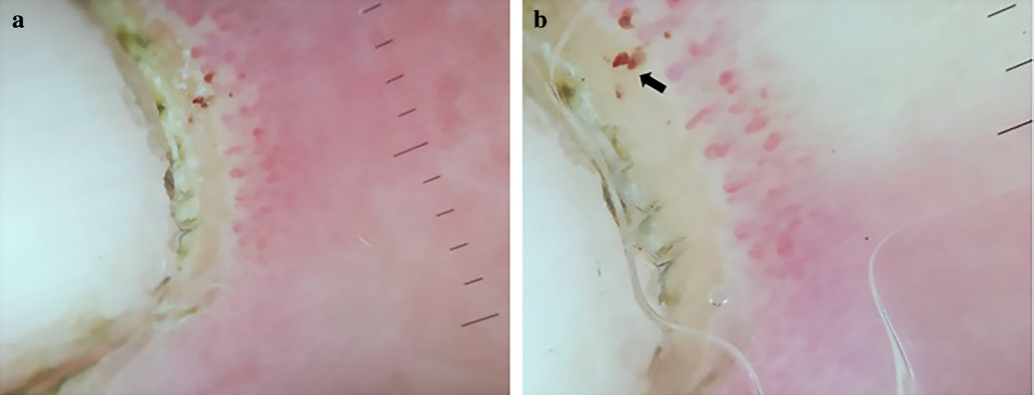
Dermoscopic findings in SLE. (**a**) Polarised dermoscopy (10 ×) of the proximal nailfold showing a diagnostic ‘SLE pattern’. Key features include dilated megacapillaries, increased loop tortuosity and multifocal microhaemorrhages. These vascular distortions and areas of capillary drop-out are highly characteristic of SLE. (**b**) Close-up polarised dermoscopy (DermLite DL5) of the nailfold showing characteristic ‘meandering’ and ‘bushy’ capillary loops. The irregular distribution and associated focal microhaemorrhages (arrows) distinguish this pattern from the more linear, ‘comb-like’ architecture typically seen in systemic sclerosis.

Dermoscopic evaluations revealed significant associations between anti-RNP levels and peripheral microvascular changes, as shown in table 4. Higher anti-RNP levels were significantly correlated with the presence of microhaemorrhage (p=0.003), capillary dropouts (p=0.021) and mild capillary dilatation (p=0.0351) (figure 3).

**Table 4:** Association between anti-RNP and dermoscopic findings

	Anti-RNP	P value
Ragged cuticle	16.8 (2.75–17.8)	0.521
Periungual erythema	26.8 (9.2–81.5)	0.235
Microhaemorrhage	28.2 (20.8–148)	**0.003**
Capillary dropouts	24.6 (13–88.5)	**0.021**
Periungual pigmentation	27.2 (6.96–52)	0.282
Mild capillary dilatation	22.3 (24.6–92.1)	**0.0351**

p value is significance if ≤0.05

Anti-RNP, anti-ribonucleic protein.

## Discussion

In a complex, multiorgan autoimmune disease such as SLE, known for its abundant autoantibody formation, immune complexes (ICs) circulation and complement system activation, microvascular changes are the hallmark of the disease, with vascular involvement considered the leading cause of death in patients with SLE.^23^ Berggren *et al* suggested that plasmacytoid dendritic cells, with their continuous production of interferon (IFN)-α, are stimulated by RNA-containing ICs, which drive the expansion of peripheral B cell subsets, creating an SLE-like phenotype.^24^ Ding *et al*, in their insightful review, stated that vascular ED is the starting point in SLE atherosclerosis, with the IFN-1 pathway being the one that mediates the dysfunction of endothelial progenitor cells in SLE atherosclerosis.^25^

This may be the unrecognised linkage we intended to explore further in our research to find a reliable, measurable marker for ED in patients with SLE. For it is prudent to recognise ED early in the process, so that more meticulous monitoring and intervention can be instituted, with the aim of preventing the development of clinically explicit microvascular complications.

### Anti-RNP and cardiac microvascular ED

It is now unanimously evident that in patients with SLE, ED is the main actor of vascular ageing and preclinical atherosclerosis during the course of the disease, contributing to the early onset of CVD and CV mortality.^4^ Patients with SLE are 9–50 times more likely to develop coronary artery disease (CAD) than the general population.^26^

We implemented the use of DSE in our test subjects because it has been well validated in many studies, with the advantages of the lack of ionising radiation and the ability to image cardiac structures and function.^27^ Initially, and based on disease activity, we observed a sharp difference in myocardial performance. In which the high-activity patients exhibited significantly lower EF (p=0.004), higher E/e (p=0.004) ratios, DPV (p<0.001) and a higher prevalence of abnormal WMSI (p<0.001) compared with the low-activity group.

This correlation between SLEDAI and diastolic dysfunction (r=0.42, p=0.008) is supported by the findings of Azoulay *et al*, who noted that systemic inflammation in SLE leads to subclinical lupus myocarditis, manifesting as increased left ventricular filling pressures.^28^ Kadoglou *et al* revealed that patients with SLE without known CVD had subtle systolic impairment compared with healthy controls, even though the classical echocardiographic parameters of systolic and diastolic function did not differ from those in healthy controls.^29^

In the current study, we observed that CFVR was significantly lower in the high-activity group (1.8±0.6) compared with the low-activity group (2.3±0.5) (p<0.001). This aligns with a prestigious review done by Manchanda *et al*, who stated that ischaemia with no obstructive CAD, which is highly prevalent in patients with SLE, is very much determined by coronary microvascular dysfunction (CMD), and is increasingly recognised as a major contributor to cardiovascular morbidity in SLE.^30^ This connection among anti-RNP, ED and the CMD is highlighted by the primary finding of our research, which is the significant negative correlation between anti-RNP antibodies, SLEDAI score and CFVR (which is considered one of the best indicators for coronary vascular bed integrity) (r=−0.30, p=0.047). This suggests that anti-RNP positivity identifies a subset of patients with SLE with impaired coronary microcirculation.

Our results align with the work of Hubbard *et al*, who demonstrated that anti-RNP antibodies are as potent drivers of interferon gene signature in SLE as anti-dsDNA antibodies via anti-RNP ICs, but without complement fixation, with the subsequent vascular activation and endothelial damage that follows the activation of the type I interferon pathway.^31^ In contrast, it is important to note that Patiño-Trives *et al* recently suggested that anti-dsDNA remains a more robust predictor of overall cardiovascular mortality.^32^

Our data entail an additional functional dimension using DSE, showing that the damage is deeper and reaches the microvascular level rather than the well-known macrovascular affection in patients with SLE. This was further proven by a recent study by Liu *et al*, who reported that the two-dimensional speckle-tracking echocardiography examination parameters can be used to predict subclinical cardiac dysfunction (by quantifying left ventricular systolic dysfunction) in patients with SLE, and anti-RNP antibodies may be an essential predictive clinical factor.^33^

### Anti-RNP and skin microvascular ED

Skin is commonly involved in SLE. The size and shape of skin lesions are determined by the intensity of vascular injury as well as the size and location of the vessels involved. The clinical manifestations include: RP, acrocyanosis, livedo reticularis, telangiectasia and cutaneous vasculitis.^23^ RP is a reversible discolouration of the digits triggered by cold exposure or emotional stress. It is found in approximately 10%–45% of subjects with SLE.^34^ One of the main pathohistological mechanisms of RP is ED, along with others like abnormal adrenergic receptor reactivity and inadequate release of neuropeptides or vasoactive mediators.^35^ Clinically, RD was more prevalent among our higher activity group. Nonetheless, 67.1% out of all our test subjects had RD, indicating an underlying overlooked link, more than just a disease flare. So we wanted to further investigate this theory by examining the integrity of the digital microvasculature using hand-held dermoscopy examination to assess NFCs, which in literature has yielded comparable intrarater and inter-rater reliability scores to nailfold video capillaroscopy, the current gold standard for detection of capillary abnormalities, with the added advantages of being more widely available and easy to use.^36^

Our results demonstrated a significant association between anti-RNP levels and dermoscopic findings and various microvascular changes in patients with SLE, such as the presence of microhaemorrhage (p=0.003), capillary dropouts (p=0.021) and mild capillary dilatation (p=0.0351). Findings that match the description of Cutolo *et al* about NFC in SLE.^37^ This association concludes that the ability of anti-RNP to stratify patients according to microangiopathic burden, reinforcing its role as an early indicator of microvascular damage, rather than a marker of established end-organ disease.

This conclusion was made clear in a study by Chebbi *et al*, who found that patients with RNP-positive SLE had more frequent giant capillaries, enlarged capillaries and ramified capillaries when compared with patients with RNP-negative SLE. They even used patients with MCTD as a control group and detected that although the decrease in capillary density was more evident in patients with MCTD, the reduction in capillary density was less severe in patients with RNP-negative SLE compared with RNP-positive SLE.^38^

### Anti-RNP and AVN

ON is generally induced by abnormal microvascular circulation, which is believed to be the result of mechanical vascular interruption, intravascular occlusion and extravascular compression.^39^ Nonetheless, multiple factors contribute to ON in SLE, including RP, gene susceptibility, lupus activity, vasculitis, hyperlipidaemia, thrombophlebitis and antiphospholipid antibodies.^40^ Also, the inflammatory burden and vascular endothelial cell injuries were suggested as risk factors.^41^

Our study showed that patients with AVN had significantly higher anti-RNP levels in their sera compared with those without AVN (median 29.8 vs 21.0, p=0.011). This goes with the results of Xiong *et al*, who found that among all autoantibodies assessed in their study, only positive anti-RNP with negative anti-Sm antibodies were significantly associated with ON. And with the now known anti-RNP link to ED, they concluded that endothelial injuries play a role in the pathogenesis of ON in SLE. They suggested that anti-RNP antibodies may be an independent risk factor.^42^

### Anti-RNP and overall disease activity

Our results showed a significant difference in serum anti-RNP levels between patients with SLE with high-activity SLE and those with low activity.^43^ Although anti-RNP antibodies are detectable in 25%–40% of patients with SLE and considered non-specific to patients with SLE, they are not typically regarded as markers of disease flares in SLE. But some previous reports have found a similar correlation between serum anti-RNP levels and disease activity.^44^ Furthermore, the presence of anti-RNP in the sera of patients with SLE at the time of diagnosis was associated with a higher median baseline SLEDAI-2K score than those without anti-RNP and a more than eightfold likelihood of experiencing a flare during the first year of follow-up.^45^

### Anti-RNP antibodies signature

The presence of anti-RNP in patients with lupus clinical features may raise diagnostic concerns. For the anti-RNP positivity remains mandatory for the MCTD diagnosis, and was always associated with certain manifestations such as RP, swollen hands/puffy fingers, inflammatory arthritis, lower risk of renal disease, myositis and lung disease.^46^ Dima *et al* suggested the use of more specific markers, such as anti-RNP or anti-Sm-D, for discriminating against SLE and MCTD. In addition, the IgM serotype of anti-RNP seems more frequently expressed in SLE, while the IgG serotype alone in MCTD.^47^

This signature was evident in our test subjects, in whom the anti-RNP levels were significantly elevated in patients presenting with skin rash and oral ulcers. And most notably, patients with myositis demonstrated markedly higher anti-RNP levels compared with those without myositis.

## Conclusion

This study highlights the significant role of anti-RNP antibodies as a multifaceted biomarker of SLE, extending beyond their traditional association with overlap syndromes. Our findings demonstrate a clear correlation between high anti-RNP titres and functional vascular impairment, characterised by reduced CFVR and distinct microvascular abnormalities. By identifying these subclinical changes, particularly in patients with high disease activity, we highlight the importance of anti-RNP in early cardiovascular risk stratification, suggesting that monitoring these autoantibodies provides vital insights into a patient’s endothelial health.

### Study strengths, limitations and future directions

The strengths of our study include the comprehensive evaluation of vascular ED using clinical, dermoscopic and advanced functional cardiac assessment, as well as correlation with immunological markers. We acknowledge that our study did not account for the ‘anti-phospholipid status’ of all patients. The absence of invasive coronary assessment and kidney biopsy limits direct histopathological validation. Longitudinal studies are needed to determine whether anti-RNP predicts progression of a widespread microvascular disease and adverse outcomes over time and whether therapeutic modulation of disease activity will alter this trajectory.

## Supplementary material

10.1136/lupus-2026-002125 online supplemental table 1

10.1136/lupus-2026-002125 online supplemental video 1

10.1136/lupus-2026-002125 online supplemental video 2

10.1136/lupus-2026-002125 online supplemental video 3

## Data Availability

Data are available upon reasonable request.
