# Supplementary material for: Old marker, new face: anti-RNP as a sentinel of coronary and peripheral endothelial dysfunction in systemic lupus erythematosus
Source: Lupus Sci Med. 2026 Jun 29;13(1):e002125. doi: 10.1136/lupus-2026-002125 (PMC13331000; doi:10.1136/lupus-2026-002125)
Supplement: online supplemental table 1 [file lupus-13-1-s001.docx]

# Supplementary table1: Comparison of anti-RNP levels according to clinical manifestations among the patients studied.

| Clinical Manifestation | Anti-RNP Median (IQR) – Absent | Anti-RNP Median (IQR) – Present | p-value |
| --- | --- | --- | --- |
| Avascular necrosis | 21.0 (6.3–27.9) | 29.8 (18.5–270.0) | 0.011 |
| Skin rash | 20.0 (4.6–27.0) | 24.6 (8.1–166.5) | 0.037 |
| Oral ulcer | 9.0 (4.6–22.1) | 26.8 (8.9–34.0) | 0.029 |
| Myositis | 21.0 (6.2–28.0) | 154.9 (29.7–282.2) | 0.010 |
| Raynaud’s phenomenon | 22.0 (9.3–29.2) | 30.2 (19.7–34.0) | 0.018 |
